# Fibrinolytic Therapy in Acute Stroke

**DOI:** 10.2174/157340310791658758

**Published:** 2010-08

**Authors:** Mònica Millán, Laura Dorado, Antoni Dávalos

**Affiliations:** Stroke Unit, Department of Neurosciences, Germans Trias i Pujol University Hospital, Universitat Autònoma de Barcelona, Spain

**Keywords:** Thrombolysis, intra-arterial thrombolysis, mechanical thrombectomy, cardioembolic stroke, stroke subtype.

## Abstract

Acute ischemic stroke is a major cause of morbidity and mortality in Europe, North America, and Asia. Its treatment has completely changed over the past decade with different interventional approaches, such as intravenous trials, intra-arterial trials, combined intravenous/intra-arterial trials, and newer devices to mechanically remove the clot from intracranial arteries. Intravenous thrombolysis with tissue plaminogen activator (tPA) within 4.5 hours of symptoms onset significantly improved clinical outcomes in patients with acute ischemic stroke. Pharmacological intra-arterial thrombolysis has been shown effective until 6 hours after middle cerebral artery occlusion and offers a higher rate of recanalization compared with intravenous thrombolysis, whereas combined intravenous/ intra-arterial thrombolysis seems to be as safe as isolated intravenous thrombolysis. The more recent advances in reperfusion therapies have been done in mechanical embolus disruption or removal. Merci Retriever and Penumbra System have been approved for clot removal in brain arteries, but not as a therapeutic modality for acute ischemic stroke since it is no clear whether mechanical thrombectomy improves clinical outcome in acute stroke. However, mechanical devices are being used in clinical practice for patients who are ineligible for tPA or who have failed to respond to intravenous tPA. We summarize the results of the major thrombolytic trials and the latest neurointerventional approaches to ischemic stroke.

Although stroke treatment has completely changed in the last fifteen years, stroke remains an important public health concern, since is the third leading cause of death in the USA, Canada, Europe and Japan and the primary cause of adult disability in these developed countries [1].

Intravenous (IV) thrombolysis with tissue plasminogen activator (tPA, alteplase) within three hours of symptoms onset is the standard of care in the treatment of acute ischemic stroke in current clinical practice. Despite of the spreading use of IV tPA in different countries and continents since 1996, IV thrombolysis has several limitations such as a short time window, a low rate of arterial recanalization, a substantial risk of intracranial haemorrhage, a moderate effect on non-selected patients and numerous exclusion criteria or contraindications which lead to a low frequency of treated patients [2]. All these facts have contributed to develop and study new thrombolytic agents, new routes of administration, longer time windows of treatment and different mechanical devices to locally remove the thrombus in the last decade.

Clinical research in this topic has elucidated that recanalization of the occluded artery is crucial in ischemic stroke treatment. A meta-analysis of more than fifty studies that evaluated spontaneous or therapeutic arterial recanalization demonstrated a strong correlation between recanalization and good outcome [3]. Recanalyzed patients had a higher probability of favourable outcome (OR, 4.43; 95% CI, 3.32 to 5.91) and a lower probability of mortality (OR, 0.24; 95% CI, 0.16 to 0.35) than non-recanalyzed patients at 90 days. Other key factor that influences on ischemic stroke outcome is time to vessel recanalization. Trials of IV thrombolysis [4,5] and also of intra-arterial thrombolysis [6] have established that good clinical outcome after successful recanalization is time-dependent. Longer times from ischemic stroke onset to initiation of treatment are associated with less absolute clinical benefit. However, delayed thrombolysis has shown to be associated with increased reperfusion/ recanalization and subsequent improved outcomes when patient selection is based on mismatch concept according to multimodal MRI or CT techniques [7,8]. 

Therefore, experimental and clinical research in acute ischemic stroke is continuously providing new strategies of acute management using pharmacological or interventional endovascular modalities and promoting the employ of radiological multimodal techniques as a treatment-selection tool. This article provides a comprehensive review of the systemic and endovascular thrombolytic treatment in acute ischemic stroke based on the largest prospective studies and randomized clinical trials (RCT) and it also reviews particular aspects of thrombolysis in cardioembolic stroke subtype.

## 1. INTRAVENOUS THROMBOLYSIS WITHIN THREE HOURS

In 1995, the pooled results of two phase III National Institute of Neurological Disorders and Stroke (NINDS) tissue plasminogen activator trials, demonstrated that IV tPA in acute ischemic stroke was safe and effective when given within 3 hours from symptoms onset avoiding patients’ death or functionally dependent of one out of seven patients treated [4]. Patients treated with tPA were at least 30% more likely to have minimal or no disability at 3 months, symptomatic intracranial haemorrhage (sHIC) occurred in 6.4% and mortality in 17% of them.

Four other phase III IV tPA trials (ECASS 1, ECASS 2, ATLANTIS A, and ATLANTIS B), did not show positive results and failed in demonstrating the benefit of tPA mainly for having enrolled a small number of patients in the under 3-hour time window. When the analysis was restricted to this period, the effect was concordant with the one found in the two NINDS trials. Despite this, a positive intention-to-treat analysis of pooled data from randomized trials of tPA for ischemic stroke was reported by Hacke *et al.* [5]. The study, that included 2,775 patients enrolled in the first 6 IV tPA trials, showed that treatment within the first 90 minutes of onset increased the odds of a favourable outcome by 2.8 fold, in the 91 to 180-minute window by 1.6 fold, and in the 181 to 270-minute window by 1.4 fold, while treatment in the 271 to 360-minute window did not improve outcome in a statistically significant way. So, the sooner tPA is given to patients, the greater the benefit is. This analysis also suggested that a longer time window was not associated with higher rates of mortality or symptomatic cerebral haemorrhage. The frequency of parenchymal hematoma was related to age and tPA administration, but neither to onset to treatment time nor to stroke severity measured by means of the NIHSS score. Consequently, the apparent reduction in benefit from tPA at later periods does not seem to be explained by an increased rate of parenchymal hematoma. 

The use of tPA for acute ischemic stroke was approved by the US Food and Drug Administration (FDA) in 1996 and subsequently by regulatory agencies in Canada (1999), South America, and Asia. In Europe, the European Medicines Agency (EMEA) approved the drug in 2002 under two conditions: (1) the implementation of an observational study (phase IV) in order to assess the use of tPA in the first 3 hours of stroke onset in clinical practice; and (2) the initiation of the third randomized clinical trial (ECASS III) to assess the effect of tPA in the 181 to 270-minute window from symptoms onset. 

The Safe Implementation of Thrombolysis in Stroke-Monitoring Study (SITS-MOST), a phase IV observational study, was started in 2002 and completed in 2007. The study included 6,483 patients from 285 different sites in 14 different countries. Half of them were centres that had not participated in any of the ECASS trials or had not enrolled more than five patients in the two ECASS trials. Primary outcomes were symptomatic intracranial haemorrhage within first 24 hours and mortality at 3 months. Secondary outcome was functional independence at 3 months (modified Rankin Scale, mRS 0-2). Compared to RCT, SITS-MOST study replicated the safety and effectiveness of IV alteplase in acute ischemic stroke in clinical practice even in poor experienced sites [9].

Ultrasound-enhanced systemic thrombolysis has been proven in tPA-treated patients in several randomized and non-randomized clinical studies [10]. According to the CLOTBUST study results, ultrasound leads to an absolute 20% increase in the rate of MCA recanalization [11]. Any recanalization was achieved in 83% of patients treated with tPA+ 2-MHz transcranial Doppler compared with 50% of patients treated with tPA alone within 2 hours of treatment, p<0.001, whereas sustained and complete recanalization at 2 hours was 38% and 13%, respectively. The sICH frequency was 3.8% in both groups. This effect was consistent with a non significant trend towards and increased rate of improved outcome in comparison with only tPA-treated patients (42% versus 29%, p=0.2). A recent meta-analysis of 6 randomized and 3 nonrandomized clinical studies of sonothrombolysis showed that any diagnostic ultrasound monitoring can at least double the chance of early complete arterial recanalization at no increase in the risk of sICH [10].

At the present time, tPA is the only approved drug that can lead to a recanalization of occluded vessels and restore brain circulation before irreversible damage has happened in order to improve the clinical outcome. 

## 2. INTRAVENOUS THROMBOLYSIS AFTER THREE HOURS

### 2.1. Intravenous Thrombolysis with Alteplase 3 to 4.5 Hours After Stroke Onset

The second condition by EMEA for tPA approval was the initiation of the third European Cooperative Acute Stroke Study (ECASS III). This randomized clinical trial assessed an extended therapeutic window beyond 3 hours. ECASS III has recently shown that patients treated with tPA in the 181 to 270-minute window had a substantially better chance to functional independence (mRS 0, 1) 3 months after treatment (52.4% vs 45.2%, OR 1.34; IC 95%, 1.02 to 1.76). Symptomatic intracranial haemorrhage occurred in 2.4% of the tPA group versus 0.2% of the placebo group, with no significant increase in mortality (7.8% versus 8.4%) [12]. 

The efficacy and effectiveness of alteplase in the 3 to 4.5-hour time window is similar to the approved 3-hour window when the drug is used in routine clinical practice. The evaluation of the Safe Implementation of Thrombolysis in Stroke-International Stroke Thrombolysis Registry (SITS-ISTR) showed that the likelihood of functional recovery, sICH, and mortality was not different between the 664 patients treated in the 3 to 4.5-hour window and the 11,865 patients treated within the first 3 hours. The rate of sICH following NINDS classification (any haemorrhage associated to neurological deterioration) was 8.5% and 8% respectively after adjustment for clinical trial prognostic factors [13]. 

ECASS III and SITS-ISTR results confirm the conclusions of the analysis of the pooled data from randomized trials of tPA for ischemic stroke [5]: IV alteplase administered between 3 to 4.5 hours after symptoms onset is effective and, despite a higher risk of symptomatic intracranial haemorrhage as compared to placebo, this treatment is as safe as given within the approved 3-hour window. The American Heart Association/American Stroke Association (AHA/ASA) guidelines for the administration of tPA following acute stroke were revised to expand the window of treatment from 3 hours to 4.5 hours in order to provide more patients with an opportunity to receive benefit from this effective therapy [14]. In Europe the recommendation has been approved in the Karolinska Stroke Update Conference (November 2008), and will be proposed for its inclusion in the European Stroke Organization (ESO) guidelines (http//www.eso-stroke.org/recommendation.php). This has not yet been FDA neither EMEA approved.

It is important to point out that, beyond 4.5 hours after stroke onset, no net therapeutic benefit has been demonstrated and a new meta-analysis of clinical trials with alteplase including data from ECASSIII suggest a significant higher risk of sICH (OR 2.96, IC95%, 1.55-5.66) when alteplase is administered in the 4.5 to 6-hour window in patients selected according to CT scan criteria [15]. 

### 2.2. Intravenous Thrombolysis within 3 to 9 Hours after Stroke Onset in Patients with Salvageable Tissue Selected by Multimodal MRI

Multiple retrospective observational clinical trials support the usefulness of multimodal MRI when selecting patients for IV thrombolysis beyond 3 hours of stroke symptoms onset. Their main findings have been: (a) MRI allows to select safely patients for thrombolytic therapy after three hours [16,17]; (b) patients with perfusion- weighted MRI (PWI) and diffusion-weighted MRI (DWI) mismatch treated with alteplase seem to be more likely to recanalize and to have less infarct volume and a better functional outcome than patients without mismatch [18-20]; (c) early recanalization of occluded artery [21] and early cerebral perfusion improvement as measured in PWI-MTT maps due to recanalization [22] are the most powerful predictors of infarct volume and functional outcome in patients treated with tPA over passing baseline parameters like mismatch volume. Some observational studies have shown that MRI-base thrombolysis with alteplase in the 0 to 6-hour window is equally or more effective than CT-based thrombolysis within 3 hours in clinical trials or in clinical practice [20, 23]. 

Several placebo-controlled clinical trials have used multimodal CT or MRI to identify and select 3 to 9-hour post-onset patients who still harbour substantial salvageable tissue and are likely to benefit from late IV thrombolytic treatment; however, this is not yet validated by a positive phase III trial. DIAS-2 results have not confirmed the favourable conclusions from the pooled analysis of DIAS and DEDAS results [24, 25]. DIAS-2 study showed a similar prevalence of favourable outcome in patients treated with desmoteplase 90 or 125μg/Kg and placebo group. The sICH rate was 4% in desmoteplase group and 0% in placebo group and mortality was higher among patients who received 125μg/Kg (21%) [26]. The possible explanations for those neutral results could be related to a less overall stroke severity, proximal MCA occlusions rate, and baseline volume mismatch compared to DEDAS and DIAS leading to higher likelihood of spontaneous recovery in the placebo group. Treatment groups were small and mortality in the high-dose desmoteplase group seems not to be related with the drug as it was not due to a higher rate of sICH. The ongoing phase III trial of IV desmoteplase (DIAS-3) that selects patients by arterial status should definitely elucidate if thrombolysis with IV desmoteplase up to 9 hours of symptoms onset is safe and effective (clinicalTrials.gov Identifier: NCT00790920).

The Echoplanar Imaging Thrombolysis Evaluation Trial (EPITHET) is a randomized, double blind and placebo-controlled clinical trial that attended to test whether alteplase given 3-6 hours after stroke onset promoted reperfusion and attenuated infarct growth in patients who had a mismatch in PWI/ DWI images. Patients were selected by CT scan criteria, randomized to tPA or placebo, and, later, and MRI was performed (not to select patients for treatment). The trial resulted negative for primary endpoint (geometric mean infarct growth) but several secondary analyses showed significant differences in favour of alteplase (median relative infarct growth was smaller and reperfusion was more common with alteplase than with placebo and was associated with less infarct growth) [27]. Alteplase was non-significantly associated with better neurological outcome (36% VS 21%) but reperfusion demonstrated to be a surrogate marker of clinical outcomes independently of recanalization [28].

Other none placebo-controlled prospective clinical trials also suggest that recanalization window could be longer in selected patients based on a penumbral pattern. In an analysis of a subset of patients from MERCI trial (mechanical embolectomy up to 8 hours from symptoms onset) studied pre-treatment and post-treatment with MRI, Kidwell *et al*. observed that response to treatment was significantly better among patients who had a penumbral pattern pretreatment. At 3 months, 89% of patients with mismatch and recanalization had a mRS 0-2 whereas it was the case of only 14% of patients without mismatch and recanalization [29]. The DEFUSE study (DWI Evolution for Understanding Stroke Etiology) has confirmed that MRI can safely identify patients who can benefit from reperfusion therapies after 3 hours and those with an unfavourable risk/benefit ratio. The authors prospectively treated 74 CT-based patients in the 3 to 6-hour time window with tPA and performed additional multimodal MRI before and after thrombolysis. This study provided unique data demonstrating that early recanalization is associated with both reduced infarct growth and better clinical outcomes in mismatch patients, but not in the absence of mismatch [30, 31].

A recent meta-analysis of data derived from 502 mismatch patients thrombolyzed beyond 3 hours (pooled from the DIAS, DIAS II, DEDAS, DEFUSE, and EPITHET trials) has been published. Their results indicate that reper-fusion/recanalization is more common amongst patients selected according to mismatch imaging who receive thrombolytic therapy (OR 3.0; 95% CI 1.6 to 5.8) and reperfusion/ recanalization is associated with improved outcomes (OR 5.2; 95% CI 3 to 9). However, delayed thrombolysis in mismatch patients did not confirm to improve clinical outcome (OR 1.3; 95% CI 0.8 to 2.0) [7]. So, delayed treatment according to mismatch selection cannot be widely recommended as part of routine care and new prospective phase III trials are required to validate the mismatch selection paradigm.

## 3. INTRA-ARTERIAL AND COMBINED THROMBOLYSIS

Intra-arterial (IA) thrombolysis consists in the local delivery of thrombolytic agents, at or within the thrombus, using neurointerventional techniques. Compared with intravenous therapy, IA therapy has the advantage of providing a higher concentration of lytic agent delivered to the clot target while minimizing the systemic exposure to drug. It has also the potential for greater efficacy with higher recanalization rates. This technique also allows the mechanical disruption of the clot with the catheter or specific devices during the procedure. Disadvantages include additional time required to initiate therapy, availability only at specialized centres, and mechanical manipulation within potentially injured vessels. Combined IA and IV thrombolysis provides the speed of initiation of IV and a trend to higher recanalization rates of isolated IA thrombolysis. Pro-Urokinase (pro-UK), urokinase (UK) and alteplase are the main thrombolytic agents used in this kind of procedures. 

Intra-arterial or combined thrombolysis has been tested only in a few controlled trials. The Prolyse in Acute Cerebral Thromboembolism II (PROACT II) study demonstrated the safety and efficacy of IA thrombolysis in patients with an MCA occlusion [32]. One hundred and eighty subjects were randomized within 6 hours of stroke onset to receive 9 mg of intra-arterial pro-urokinase (pro-UK) and heparin (n=121) or IV heparin alone (n=59). Patients in the pro-UK group had a greater recanalization rate (partial or complete: 66% versus 18%, p=0.001; complete: 19% versus 2%, p=0.004) and a better functional outcome defined as a mRS 0-2 at 3 months (40 versus 25%, p=0.04) than patients in the control group. Although sICH rate was greater in the pro-UK group (10% versus 2%, p=0.06), overall mortality rates were similar in the two treatment arms (25% versus 27%). The Middle Cerebral Artery Embolism Local Fibrinolytic Intervention Trial (MELT) investigated IA urokinase up to 6 hours after stroke onset in patients with a MCA occlusion (M1 or M2) [33]. One hundred and fourteen subjects were randomized to placebo or intra-arterial UK until the study was stopped when IV alteplase was approved in the 0 to 3-hour window. Despite the study was non-positive on its primary end point of the proportion of patients with good functional outcome (mRS 0-2 at 3 months), a favourable trend was perceived (49.1% versus 38.6%, p=0.345). However, for the secondary end point of excellent functional outcome (mRS 0-1 at 3 months), substantial benefits were observed (42.1% vs 22.8%, p=0.045). A meta-analysis of both IA fibrinolysis trials (PROACT and MELT) supported the benefit of IA fibrinolytic therapy in MCA occlusion within 6 hours of symptoms onset [34]. 

Even though the FDA and international regulatory agencies did not approve a stroke labelling for pro-UK with a single, small, phase III clinical trial, IA thrombolysis therapy is commonly administered as an off-label therapy for stroke at tertiary centres within 6 hours of onset in the anterior circulation and up to 12-24 hours after onset in the posterior circulation. ESO 2008 & AHA/ASA 2007 guidelines recommend intra-arterial treatment of acute MCA occlusion within a 6-hour time window as an option (Class II-I, Level B) and in patients with contraindications to the use of IV thrombolysis, such as recent surgery (Class II, Level C).

No RCT have compared IA and IV thrombolysis in acute ischemic stroke. Although some indirect data suggest a higher rate of recanalization of the IA way, it is no clear whether the longer time spent in the IA procedure counterbalance the potential benefit of IA thrombolysis. Combined intravenous and intra-arterial thrombolysis is another thrombolytic strategy investigated in several non-controlled trials that has the benefit of faster initiation of IV treatment followed by rescue IA revascularization in patients who have not had a successful recanalization after IV treatment and higher recanalization rates than IA approaches. Alteplase administered intra-arterially and intravenously was the drug used in largest and latest studies in that field. The Emergency Management of Stroke Trial (EMS) and the Interventional Management of stroke Trial (IMS) demonstrated that the combined IV/IA approach had similar rates of mortality and sICH compared with subjects of similar severity and age treated with IV tPA alone in the NINDS stroke trial [35, 36]. IMS study included patients younger than 80 with a NIH Stroke Scale ≥10. Combined IV tPA (0.6mg/kg, 60mg maximum over 30 minutes) started within 3 hours of onset with additional tPA administered via micro catheter at the site of the thrombus (up to a total dose of 22 mg over 2 hours or until thrombolysis) was safe (6.3% of sICH in IMS compared to 6.6% in tPA treated patients of NINDS) although good functional outcome was similar to tPA treated patients of NINDS (43% versus 39%). IMS II results have been recently published [37]. The IMS II study protocol was the same than the IMS trial but, in that case, intra-arterial tPA was administered via the EKOS micro-infusion catheter or a standard micro-catheter. The EKOS micro infusion catheter uses acoustic streaming to increase fluid penetration, thus driving the thrombolytic agent into the thrombus. Recanalization rate was higher than in IMS I (73% versus 56%), but there were not statistically significant differences with respect to functional outcome at 3 months (46% versus 43%). IMS III is now under way. This phase III, randomized and open label trial is testing standard IV tPA treatment alone with combined therapy (lower-dose IV tPA plus one of a few intra-arterial treatments) in patients in whom tPA is initiated within 3 hours of acute ischemic stroke onset (ClinicalTrials.gov Identifier: NCT00359424).

## 4. MECHANICAL THROMBOLYSIS

Mechanical thrombolysis involves the use of catheters to directly deliver a clot-disrupting or retrieval device to a thromboembolus that is occluding a cerebral artery. This approach can improve the rate and speed of recanalization and could decrease the incidence of intracranial haemorrhage compared with intra-arterial pharmaceutical lytics. Case reports and small case series suggested that the use of intravascular devices for clot removal in the endovascular treatment of ischemic stroke can be considered for patients who are not thrombolytic candidates, such as those that have had a recent surgical procedure or are under anticoagulation treatment [38, 39]. 

First patients treated as part of the Mechanical Embolus Removal in Cerebral Ischemia (MERCI) multicenter safety trial were published in 2004 [40]. The thrombus retrieval device employed (MERCI retriever) is a flexible nitinol wire with coil loops that is used with a micro catheter and a balloon-guided catheter. Twenty-eight patients with contraindication to IV thrombolysis were included in the trial within an 8-hour window. This phase I study showed that successful recanalization (TIMI grades 2 or 3) with mechanical embolectomy was achieved in 12 (43%) patients and with additional intra-arterial tPA in 18 (64%). No symptomatic intracranial haemorrhage occurred but overall efficacy and safety achieved with the MERCI retrieval system were not superior to those occurred with IA Pro-UK in the PROACT II study. Full MERCI trial results were published in 2005 [41]. This trial provided data about the safety and effectiveness of endovascular embolectomy with Merci Retriever in restoring vascular patency during an acute ischemic stroke and provided an alternative intervention for patients ineligible for IV thrombolysis. The device was deployed in 141 of 151 patients included in the study and additional tPA was administered in 51 patients. Baseline median NIHSS score was 20. Recanalization with the device was achieved in 68 patients (48%) but additional adjuvant therapy (tPA, UK, angioplasty, snare) led to recanalization in 85 (60%) subjects. The overall of good outcome (mRS ≤ 2) rate was 27.7%. Symptomatic intracranial haemorrhage was observed in 11 of 141 (7.8%) patients and clinical significant procedural complications (vascular perforation, intramural arterial dissection or embolization of a previously uninvolved territory) occurred in 10 of 141 (7.1%) patients. Mortality rate in the 141 patients in whom the device was deployed was 44%. Importantly, successful recanalization was associated with good neurological outcomes (46% versus 10%) and reduced mortality (32% versus 54%) and was the strongest predictor of favourable outcome at 3 months (OR, 12.82; 95% CI 2.95 to 55.75). 

Based on these results, in 2004, the FDA approved the Merci Retrieval System to remove clots from vessels in patients experiencing an ischemic stroke ineligible for intravenous thrombolytics or as a rescue therapy after unsuccessful recanalization with tPA. After that, the Multi MERCI trial was designed to further evaluate the safety and efficacy of combining IV tPA with mechanical thrombectomy and to test a newer-generation thrombectomy device (L5 Retriever) compared to first-generation device [42]. This study included patients within 8 hours of stroke onset who had either failed to respond to IV tPA or were ineligible for IV tPA but were still eligible for IA treatment. Baseline median NIHSS score was 19. Twenty-nine percent of patients received IV tPA prior to treatment with the Merci Retriever (0.6-0.9mg/Kg) and recanalization was achieved in 55% (90/164) of patients with the device alone and increased to 68% (112/164) with combined mechanical and IA thrombolytic therapy. Symptomatic intracranial haemorrhage occurred in 16 patients (9.8%), 5/45 (10%) of those treated with combined use of IV tPA and MERCI retriever and 11/116 (9.5%) in those that did not receive tPA. Overall, clinically significant procedural complications occurred in 5.5% and mortality in 34%. A favourable outcome, modified Rankin score of ≤ 2, was seen in 36% (59/164) of patients at 90 days. The overall rates of recanalization (68%), good outcome (36%) and mortality (34%) were substantially improved in comparison with those in the MERCI trial. Again, outcomes trended better in those patients in whom the vessel opened compared with those in whom it did not with lower mortality (25% versus 52%) and higher good clinical outcomes (49% versus 9.6%). These results allowed to conclude that mechanical thrombectomy after IV tPA seems as safe as mechanical thrombectomy alone and that the newer generation thrombectomy devices achieves higher recanalization rates compared with the first-generation devices. 

A number of mechanical thrombolysis devices have entered clinical trials for treatment of acute stroke. The FDA approved the Penumbra System in 2007 to open vessels in patients with ischemic stroke. The device uses aspiration to remove the clot. The initial safety trial included 23 patients with cerebral ischemia up to 8-hours after symptoms onset [43]. All of the treated vessels (100%) were successfully recanalized (TIMI 2 or 3). At 30-day follow-up, 45% of patients had a mRS <3 or a 4-point or more NIHSS score improvement. Mortality rate was 45% taking into account that 70% of the subjects at baseline had either an NIHSS score of more than 20 or a basilar occlusion. A study assessing the safety and efficacy of the system for recanalization of vessels was completed in the United States and Europe [44]. The goal was to prove “substantial equivalence” to the MERCI device in opening occluded cerebral blood vessels in stroke. The Penumbra System trial treated a total of 125 target vessels in 125 patients. Patients must present within 8 hour of symptoms onset, with neurological deficits as defined by an NIHSS score ≥ 8 and an angiografically verified occlusion of a large intracranial vessel. Patients presenting within 3-hour window not eligible for IV tPA or with a persistent vessel occlusion after IV thrombolysis could be treated. The baseline median NIHSS score was 18. The recanalization rate was 81.6% (TIMI 2 or 3). Symptomatic intracranial haemorrhages occurred in 11.2%. All cause mortality was 32.8% at 90 days with 25% of patients achieving a modified Rankin score ≤2. Similar to the MERCI and MultiMERCI trials, good outcome were more frequent (29% versus 9%) and mortality rate was lower (29% versus 48%) with successful compared with unsuccessful recanalization. 

In summary, both devices (MERCI retriever and Penumbra System) can safely and effectively revascularize large intracranial vessels in patients experiencing ischemic stroke that present within 8 hours from symptoms onset although whether such revascularization leads to a better functional outcome compared to medical management alone has not been still demonstrated.

A systematic review and meta-analysis of different studies in which mechanical thrombectomy with diverse devices have been used in the treatment of ischemic stroke was reported in 2008 [45]. The pooled cohort was compared with a historical cohort matched for sex, age, and NIHSS score. The search yielded 114 publications with 298 included patients, but only 143 were analyzed with a mean NIHSS score of 20. The clot was accessible in 85% of the patients. Any intracranial haemorrhage occurred in 22% of the patients. Of 81 patients with concurrent thrombolysis, 18.5% had ICH compared with 27.3% of 66 patients without thrombolysis (p=0.21). Of the 126 patients with accessible clots, 36% had a good mRS (2) and 29% died, whereas in patients with inaccessible clots, 24% had a good mRS and 38% died. Factors associated with clinical success were younger age (p =0.001) and lower NIHSS score at admission to the hospital (p=0.001). Compared with a matched cohort, patients who received mechanical intervention were 14.8 times more likely to have a long-term good outcome. There were no differences is the safety parameters between devices, except a trend to higher long-term functional independence with snare device. 

Endovascular therapy as a treatment of acute ischemic stroke is under incessantly investigation. Recent prospective clinical studies have shown that mechanical approaches combined with intra-arterial pharmacological therapy are associated with higher recanalization rates than either intervention alone (87.7% combined versus 17.6% intra-arterial lytics alone versus 46.2% mechanical alone) in acute internal carotid artery terminal occlusion [46], and that delayed endovascular recanalization later than 8 hours after symptoms onset can be safe and effective in carefully selected patients by multimodal MRI/CT techniques [47, 48].

However, the true effect on the clinical outcomes of these patients would only be elucidated through a randomized trial. The Magnetic Resonance and Recanalization of Stroke Clots Using Embolectomy (MR RESCUE) trial is currently testing the effects of embolectomy with MERCI Retriever stratified by the presence of MRI mismatch versus non-mismatch pattern at randomization. This randomized trial funded by NINDS compares the mechanical approach with medical therapy in the 8-hour window from symptom onset. Participants are randomized to receive treatment either with the Merci Retriever and standard medical care or standard medical care alone (clinicalTrials.gov Identifier: NCT 00094588).

No other mechanical thrombolysis devices have received FDA clearance besides MERCI Retriever and Penumbra System. The EKOS ultrasound device is currently being evaluated in the IMS III clinical trial as one of the intra-arterial interventions in the combined IA/IV group approach. The Ultrasound Thrombolytic Infusion Catheter combines the use of a distal ultrasound transducer with infusion of a thrombolytic agent through the micro catheter. In a pilot study, 14 patients with acute stroke (5 anterior circulation occlusions and 4 posterior circulation occlusions) were treated with IA tPA or rateplase infusion through the EKOS micro catheter [49]. The median NIHSS score was 19.5 (range, 9-27). TIMI grade 2-3 was achieved in 8 (57%) subjects in the first hour with an average time to recanalization of 46 minutes. No adverse events related to the catheter were registered. Symptomatic ICH rate was 14% and mortality rate was 36%. Forty-three percent of patients were functionally independent (mRS≤2) at 90 days follow-up. As a result, the device was used in 33 of 81 patients enrolled in the IMS II trial. Recanalization rate was higher when EKOS catheter was employed (24/33, 73%), compared to standard micro catheter used in IMS I trial (33/59, 56%).

The experience is limited in other mechanical approaches. A few devices have been tested in clinical trials that had to be discontinued, some because of financial considerations and other for safety reasons. A number of mechanical thrombolysis devices (such as Snarelike devices or Suction thrombectomy devices) have not still been assessed in clinical trials.

## 5. THROMBOLYSIS IN CARDIOEMBOLIC STROKE

Cardioembolic stroke accounts for one third of all ischemic strokes, and atrial fibrillation is the cardiac source of emboli in 50% of them [50]. Anticoagulation as a secondary prevention treatment of cardioembolic stroke subtype has outstandingly reduced the annual risk of stroke in these patients and has completely changed their long-term survival [50]. However, acute cardioembolic stroke is associated with high morbidity and mortality since it usually causes a severe baseline neurological impairment, large infarct volumes and an increased risk of hemorrhagic transformation, above all when delayed spontaneous or pharmacological-induced arterial recanalization occurs [51-53].

The rationale for thrombolysis for acute ischemic stroke is recanalization of occluded arteries to re-establish brain function by saving tissue at risk. Intravenous thrombolysis clinical trials clearly demonstrated a beneficial effect of IV tPA when given < 4.5 hours after symptoms onset [4, 5, 12]. The efficacy of IV thrombolysis has been established in non-selected patients because there was no interaction between tPA treatment and different baseline variables, suggesting a persistent beneficial effect of tPA across all subgroups of patients, even for all stroke subtypes [54]. However, these IV thrombolysis clinical trials did not monitor presence and location of arterial occlusion and recanalization at different times after stroke.

Arterial recanalization in acute stroke is associated with higher probability of long-term good outcome and lower probability of mortality [3]. Intravenous thrombolysis and pharmacological and mechanical intra-arterial thrombolysis have demonstrated to achieve higher and earlier vessel recanalization rates than spontaneous recanalization in acute stroke [32, 55, 56]. Successful recanalization also depends on the site and extent of the thromboembolic occlusion being markedly lower in patients with intracranial internal carotid occlusion than in those with occlusions of the M1 or M2 segments of the middle cerebral artery [57, 58]. However, there are few data about the influence of the thromboembolus type on arterial recanalization and, in consequence, about the response to thrombolytic therapy among different stroke subtypes. 

Experimental studies have revealed that lytic susceptibility and penetration of thrombolytic agents into the thrombus depends on the specific structural aspects of clots. Old, platelet-rich, and well organized thrombi formed under flow conditions have been shown to be more resistant to thrombolysis than fresh, fibrin- and red cell-rich clots formed under conditions of stasis [59, 60]. In humans, thromboembolic arterial occlusions may originate from various proximal sources, including venous sites, mural cardiac thrombi, or atherosclerotic lesions within or proximal to the affected vessel. Cardiac source of clot might probably represent the stroke subtype with more uniform fibrin-rich clots and higher efficacy of thrombolysis. A clinical study in 72 patients with proximal middle cerebral artery occlusion treated with IV tPA within 3 hours of onset showed a differential pattern of tPA-induced arterial recanalization among stroke subtypes [61]. Early recanalization during tPA infusion was more frequent in patients with cardioembolic stroke (59%) compared with large-vessel disease (8%) and undetermined origin (50%). Moreover, recanalization was significantly more complete (50% versus 27%) and faster in cardioembolic stroke compared with other stroke subtypes. Importantly, a graded response in neurological improvement at 24 hours and long-term outcome was observed in relation to the speed of clot lysis during tPA administration and consequently, favourable outcome at 3 months was higher (59% versus 40% versus 11%) and mortality rate was lower (13% versus 19% versus 41%) in cardioembolic stroke patients in comparison with undetermined and large-vessel disease stroke, respectively [61]. Middle cerebral artery recanalization rate has been also confirmed to be higher in cardioembolic stroke in an intra-arterial thrombolysis clinical study in which 76 patients were treated within 6 hours of symptom onset [51]. Complete recanalization was achieved in 83% of cardioembolic and 61% of atherosclerotic strokes although in this study poor outcome and mortality (mRS >2) were more frequent in cardioembolic stroke (50% versus 35%), probably for the reason that neurological deficit at admission was more severe in cardioembolic (mean NIHSS 19.7) than in atherothrombotic (mean NIHSS 16.6) subtype [51]. The trend to a higher rate of vessel recanalization in cardioembolic stroke with thrombolytic therapies might be as well explained for low plasma levels of endogenous tPA and its inhibitor (plasminogen activator inhibitor-1 or PAI-1) since the raise of these markers have been related to increased risk of atherothrombotic ischemic events such as stroke or myocardial infarction [62]. Nevertheless, tPA antigen, PAI-1 antigen, PAI activity and tPA/PAI-1 complex levels were significantly higher in ischemic stroke patients in comparison with a control group, but no differences were found regardless of stroke etiology except for lower levels of PAI-1 antigen in cardioembolic stroke in a case-control clinical study [63]. 

There are some studies that are not in line with a different pattern of recanalization according to stroke subtype. In a local intra-arterial thrombolysis study with UK in 62 patients with middle cerebral artery or intracranial internal carotid occlusion, only the thromboembolus location affected arterial recanalization. Neither stroke etiology nor other baseline parameters were related to successful recanalization [58]. Importantly, within the group of cardioembolic occlusions, recanalization was significantly less when transoesophageal echocardiography showed a cardiac thrombus compared with those patients in which did not reveal the thrombus, probably explained by the different composition and age of the clot [58]. The embolic material is likely to be fibrin-rich in patients with atrial or ventricular thrombus, whereas in other conditions such as patent foramen ovale, right-to-left shunt, venous thrombi or recent onset of arrhythmia, clot is supposed to be fresher with higher content of platelets and red blood cells leading to an easier recanalization with thrombolytic therapies. Histological analysis of thromboembolus retrieved by endovascular mechanical extraction from the middle cerebral artery and intracranial carotid artery in 25 patients with acute ischemic stroke revealed that most clots (75%) shared architectural features of random fibrin-platelet deposits interspersed with linear collections of nucleated cells and confined erythrocyte-rich regions, being red clots composed uniquely of erythrocytes and cholesterol crystals uncommon [64]. In addition, cardioembolic and atherosclerotic sources of embolism had similar histological components with a high prevalence of a fibrin:platelet pattern [64]. 

Hence, it is controversial whether vessel recanalization with IV or endovascular thrombolytic therapies depends on the characteristics of the clot, and consequently, on the stroke subtype [51, 58, 61, 63, 64]. However, results of large clinical studies [64] and main randomized clinical trials of IV thrombolysis [4, 5, 54] have demonstrated no significant difference in final outcome in tPA-treated patients based on confirmed stroke mechanism. 

## 6. CONCLUSIONS

Since 1996 after tPA approval, treatment of acute ischemic stroke has totally changed. Intravenous thrombolysis has demonstrated to be safe and effective up to 4.5 hours after symptoms onset, however the frequency of treated patients is still quite low due to a number of burdens and failures in the optimal accomplishment of the treatment. Endovascular therapy in acute ischemic stroke has become a promising alternative for patients who are ineligible for intravenous thrombolysis or have failed in recanalyzing the occluded artery. Neurointerventional approaches in the treatment of acute ischemic stroke offer higher recanalization rates compared with intravenous thrombolysis but it is no clear whether improve outcomes. Randomized clinical trials are necessary to elucidate the true effect of endovascular thrombolysis on clinical outcomes.

## Figures and Tables

**Table I:** Baseline Stroke Severity and Outcome Variables in the Main Endovascular and Intravenous Thrombolytic Trials

	n	NIHSS Basal	Successful Recanalization (TIMI 2-3)	mRS 0-2 at day 90	90-Day Mortality	sICH
**Intra-arterial thrombolysis**						
PROACT II [29]	121	17	66%	40%	25%	10%
MELT [30]	114	14	74%	49%	5%	9%
IMS [33]	62	18	56%	43%	16%	6%
IMS-II [34]	55	19	58%	46%	16%	10%
**Mechanical thrombectomy**						
MERCI [38]	141	20	48%+/60%	28%	44%	8%
Multi MERCI [39]	164	19	55%+/68%	36%	34%	10%
Penumbra [41]	125	18	82%+	25%	33%	11%
**Intravenous thrombolysis**						
Pooling analysis of IV tPA trials within 6 hours (tPA) [5]	1391	11	NA	49%	13%	5-9%*
**Control groups**						
PROACT II (control) [29]	59	17	18%	25%	27%	2%
MELT (control) [30]	57	14	NA	39%	3.5%	2%
Pooling analysis of IV tPA trials within 6 hours (placebo) [5]	1384	11	NA	44%	15%	1.1%*

sICH: symptomatic intracerebral hemorrhage

+ Device alone;

* Parenchymal hematoma type II

tPA: tissue plasminogen activator, mRS: modified Rankin Scale; NIHSS: National Institutes of Health Stroke Scale.
